# A Gentleman

**Published:** 1879-09

**Authors:** 


### A GENTLEMAN

is not made, but born. The artificial article—man or woman—is a sham, a pretence, a got up affair for the occasion. Only let self-interest be invaded, especially when superiors in position are not present, and how soon the mask falls of; and unbridled anger, and vulgar epithet, and wrathful scowl of countenance write high on the forehead, "Gutter," "Coventry," as their origin and destination.

The gentleman has drawn from his mother's milk a high sense of honor; a punctiliousness of right and wrong in small matters and large; adherence to all that is courteous, and pure, and true; he is controlled by a lofty sense of principle in all business transactions, in all companies, and in every emergency. The polish, and gentleness, and integrity, and courtliness of a born gentleman show out as the pure sparkling waters of an over-welling spring; are not shot off with the mechanical precision of deliberately pointed artillery. In shorter phrase, the gentle man and gentle lady are born with hearts generous, and good and true.

